# The Study of Zinc Ions Binding to α_S1_-, β- and κ-Casein

**DOI:** 10.3390/ijms21218096

**Published:** 2020-10-30

**Authors:** Agnieszka Rodzik, Paweł Pomastowski, Viorica Railean-Plugaru, Myroslav Sprynskyy, Bogusław Buszewski

**Affiliations:** 1 Department of Environmental Chemistry and Bioanalysis, Faculty of Chemistry, Nicolaus Copernicus University, Gagarina 7, 87-100 Toruń, Poland; agnieszka.rodzik1@gmail.com (A.R.); rviorela@yahoo.com (V.R.-P.); mspryn@chem.umk.pl (M.S.); bbusz@chem.umk.pl (B.B.); 2 Centre for Modern Interdisciplinary Technologies, Nicolaus Copernicus University, Wileńska 4, 87-100 Toruń, Poland

**Keywords:** α-casein, β-casein, κ-casein, zinc ions, sorption, kinetic, FT-IR, Raman, MALDI-TOF MS

## Abstract

The presented studies focused on the specificity binding of particular casein fractions: α_S1_-, β- and κ-casein (α_S1_CN, βCN, κCN), with zinc ions. The binding mechanism was determined by kinetic modeling using results of batch sorption. For this goal, models of zero-order kinetics, pseudo-first-order, pseudo-second-order and Weber–Morris intraparticle diffusion were used. The formation of Zn-α_S1_CN, Zn-βCN and Zn-κCN complexes was additionally monitored using spectroscopic methods such as Fourier transform infrared spectroscopy (FT-IR) and Raman spectroscopy, characterizing active functional groups involved in the binding process. Additionally, a mass spectrometry technique—matrix-assisted laser desorption/ionization time-of-flight mass spectrometry (MALDI-TOF MS)—was used to characterize respective protein fractions and obtained complexes. Spectroscopic and spectrometric studies were carried out both before and after binding the protein with zinc ions. The obtained results showed the difference in Zn-α_S1_CN, Zn-βCN and Zn-κCN complexes created at separate kinetic stages. On the basis of instrumental studies, a significant influence of acidic (glutamic acid (Glu), aspartic acid (Asp)) and aromatic (tryptophan (Trp), phenylalanine (Phe), tyrosine (Tyr)) amino acids on the formation of metal complexes was proven. In turn, spectrometric studies allowed determining the molecular masses of casein isoforms before and after binding to zinc ions.

## 1. Introduction

Casein is recognized as the main cow’s milk protein, accounting for about 80% of the total milk protein content [1]. It is not a homogeneous protein but consists of several fractions. The main fractions are α (α_S1_, α_S2_)-, β- and κ-casein (αCN, βCN and κCN). They form colloidal aggregates called micelles in combination with calcium phosphate [2]. The primary role of caseins in milk is to ensure the effective transport of calcium and phosphate from the mammary gland directly to the newborn baby [3]. The main components of casein (αCN, βCN and κCN) form a strong micellar complex stabilized by van der Waals forces, hydrophobic effects, hydrogen bonding and electrostatic and steric stabilization [2]. In addition, micelles are stabilized by physicochemical properties such as the ability of phosphorylated serine residues to bind to calcium ions and the amphiphilic nature of κCN, which is a fraction that is glycosylated and responsible for stabilizing micelles on the surface [4]. The size of micelles varies depending on the amount of specific fractions—mainly κCN—cow feed and season (in summer, the micelles are smaller, and in winter, the opposite is true) [5]. The amount of calcium ions is also important in forming stable micelles [5]. In addition, during casein hydrolysis, caseinophosphopeptides are formed, which bind calcium through their phosphoserine residues, affecting casein stability [6]. It turns out that caseins have a natural ability to bind to other metal ions such as silver [7], iron [8] and zinc [9,10]. Despite this knowledge, there are still many uncertainties in the mechanism of binding metal ions to casein fractions, especially their quantitative contribution and the role of the active functional group in this process.

Fraction α_S1_CN accounts for 40% overall casein in bovine milk. It is a single-chain polypeptide with a sequence containing 199 amino acid residues containing sixteen serine (Ser) residues, eight of which are phosphorylated and seven of which occur between 43 and 80 residues [11]. Furthermore, this highly acidic segment also contains twelve carboxyl residues and is responsible for the negative charge of the molecule [4,11]. As a result of negative charges at neutral pH, phosphoric residues and secondary carboxylic acid residues have a strong affinity for metals [9]. The second significant fraction in the total amount of caseins in milk is βCN. βCN is a single-chain polypeptide with five residual phosphoserines with the first four forming the phosphorylation center [4,11]. It represents about 35% of bovine milk casein content and consists of 209 amino acids residues. βCN is strongly amphipathic and the N-terminal part of the βCN molecule (1–43 residues) contains a negative protein charge, has low hydrophobicity and consists of only two prolines (Pro) residues, representing about 17%. The βCN central section, i.e., residues 44–135, has a low charge and moderate hydrophobicity, while the C-terminal part of the molecule (136–209 residues) contains many non-polar residues and is therefore highly hydrophobic [4]. κCN is the third main but smallest casein protein (169 residues) with a low calcium sensitivity. It is the only form of casein that can be glycosylated in addition to phosphorylation [4]. Phosphorylation compared to αCN and βCN can also take place in the rest of the threonine (Thr). Thr takes part in binding with zinc ions [9]. Due to the considerable distances between residues of SerP and ThrP κ-casein, there is no phosphorylation center [4]. 

The structure of casein micelles most probably consists of a form of αCN and βCN located in the inside of the micelles and κCN forming the outside layer stabilizing the micelles sterically [12]. The stabilization of micelles is possible thanks to the hydrophilic part of κCN protruding into an aqueous surrounding—glycomacropeptide. The actual internal structure of casein micelles is not fully understood and explained [13]. For this reason, several models were developed to characterize casein micelle [14]. The main models proposed are (i) the submicellar (subunit) model [15], (ii) the coat-core model [16] and (iii) the internal structure model [17].

The colloidal nature of milk poses a great challenge to isolate proteins. Casein micelles and fat globules function as separate phases, preventing milk filtration and complicating the usual separation methods [18]. Since purified individual milk proteins have better functionality than their native protein mixtures, there is great interest in developing easier methods of preparing pure casein and its isoforms on a large scale [2]. To eliminate protein–protein interactions, different concentrations of urea [19], dimethylformamides [20], β mercaptoethanol [21] or dithiotreitol [19] are used, among other things, to change the structure of proteins by splitting hydrogen bonds and reducing disulphide bonds. Differences in αCN, βCN and κCN solubility in urea solution allow for separating different components [2]. In addition, the combination of proteins with d-electron metal ions allows for obtaining nanocomposites such as metallocomplexes (with zinc ions [9], uranium(VI) [22]) and nanoparticles (with silver ions) [23] with a wider spectrum of biological activity. The mechanisms by which a protein binds metal ions, which often improve protein function, are not well known [24]. 

Zinc ions are essential for many living systems, playing a significant role in physiological reactions and diseases [25]. Zinc is a structural and catalytic component of many proteins. It modulates the functions of glutamate and neurotransmitter receptors, regulates transcription factors and inhibits tyrosine phosphatase proteins [26]. This element is also essential for the functioning of many enzymes. Its deficiency contributes to congenital defects and acquired immunological responses [27]. Understanding the mechanism of zinc ion binding to casein and structural changes in protein induced by these ions can prevent immune deficiencies or disease changes [10]. The use of knowledge about the mechanism of binding zinc to casein can be a tool in their medical application [28]. Pomastowski and co-authors studied the interaction of zinc ions with casein [9], however, the distribution of the zinc ion binding in various casein fractions was not known. 

Hence, the main goal of this study was to explain the binding process of zinc ions to individual casein fractions. Therefore, models of zero-order kinetics, pseudo-first-order, pseudo-second-order and Weber–Morris intraparticle diffusion were applied. This objective has been achieved by previous characterization of individual casein fractions (α_S1_CN, βCN, κCN) isolated from cow’s milk, and the study of their dispersion stability, isoelectric point and molecular masses. In addition, spectroscopic (FT-IR, Raman) and spectrometric (MALDI-TOF MS) studies were carried out to determine the contribution of the active functional group in the binding process of zinc ions to casein fractions. 

## 2. Results and Discussion

### 2.1. Characteristics of α_S1_CN, βCN, κCN

**Figure 1:**
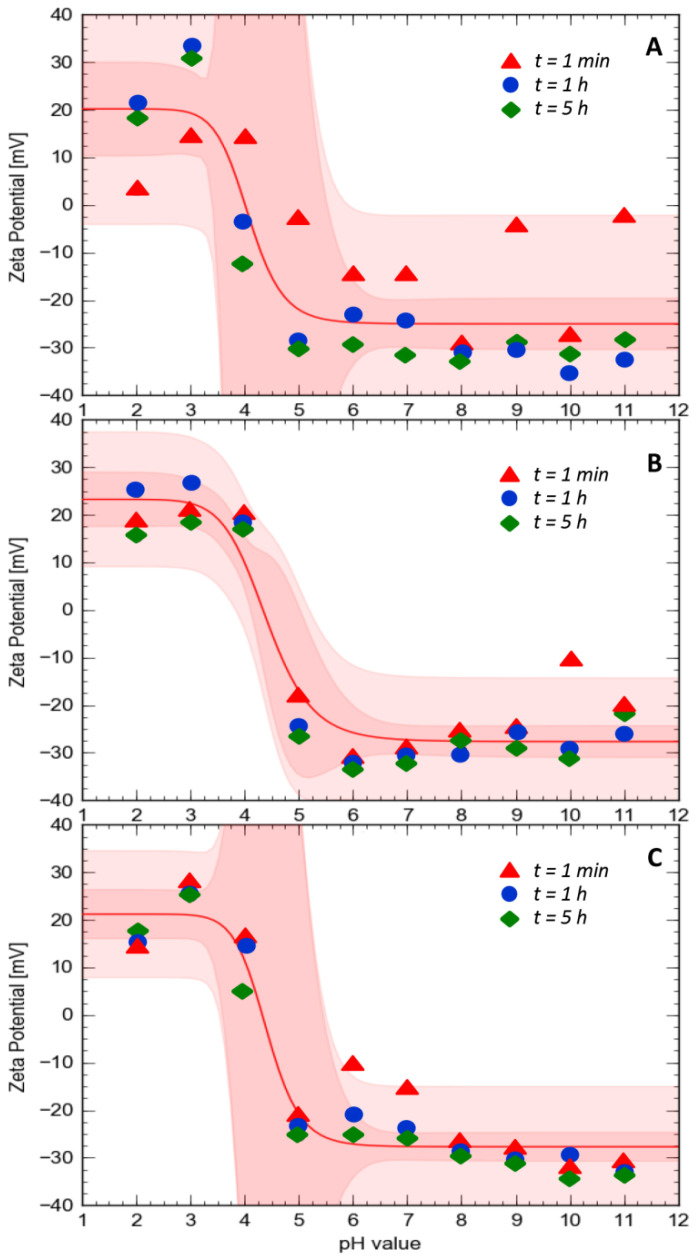
Zeta potential of α_S1_CN (**A**), βCN (**B**) and κCN (**C**) as a function of pH. The red line represents the sigmoidal fit trend line. Darker red indicates a confidence band, while lighter indicates a prediction band.

In order to determine the isoelectric point and to examine protein stability, the zeta potential value was measured for the initial time (t = 1 min), after one hour (t = 1 h) and after five hours (t = 5 h). The zeta potential results of α_S1_CN, βCN and κCN as a function of pH (2–11) are presented in Figure 1. Isoelectric points (pI) for α_S1_CN, βCN and κCN were found to be 4.80 ± 0.72, 4.55 ± 2.15 and 4.40 ± 0.28, respectively. The surface charges of all investigated proteins are in the range of −35–+32 mV. In addition, it was observed that with increasing time, the zeta potential values changed and protein stability was noticed after t = 1 h and t = 5 h. At low pH values, the surface charge of proteins was positive and the zeta potentials became more negative with increasing pH. The results show that the more stable proteins are βCN and κCN and the least stable is α_S1_CN. The potential values of α_S1_CN, βCN and κCN at pH = 2 and pH = 3 were found between +15 and +32 mV, except t = 1 min for α_S1_CN, for which the zeta potential was noticed between +3 and +14 mV. Above pH = 3, a decrease in the zeta potential to −20/−30 mV at pH = 5 for each protein was observed. In the case of t = 1 min for α_S1_CN, the decrease in the zeta potential occurred from pH = 4 to pH = 6. 

The zeta potential provides an indicative measure of dispersion stability. The chemical properties of the surface of the particles affect the zeta potential of each dispersion. The surface chemistry can be modified by changing the pH, surfactant concentration and salt concentration. Therefore, it is important to determine the effect of pH on the zeta dispersion potential [29].

The protein charge is mainly controlled by two processes: (de)protonation of functional surface groups and counter-ions condensation on the protein surface [30]. Farrel et al., 2004 [31], indicated that pI for α_S1_CN, βCN and κCN were 4.92–5.05, 5.41 and 5.77, respectively, while according to Egito et al., 2002 [32], the pI values for α_S1_CN, βCN and κCN were 4.4–6.3, 4.4–5.9 and 3.5–5.5, respectively. The pI depend on the origin of the sample, the method of determination and the used electrolyte [23].

### 2.2. Kinetic Study of the Zinc Binding Process

Kinetic studies contribute to the understanding of the mechanism of binding casein isoforms (α_S1_CN, βCN and κCN) with zinc ions. The obtained experimental kinetic data were examined with reference to zero-, pseudo-first- and pseudo-second-order kinetic models and the Weber–Morris intraparticle diffusion model. Matching the experimental kinetic data to the models allowed for explaining the rate of the binding of zinc ions to proteins, but also for determining the degree of adsorption of zinc ions on the protein adsorbent. 

**Figure 2:**
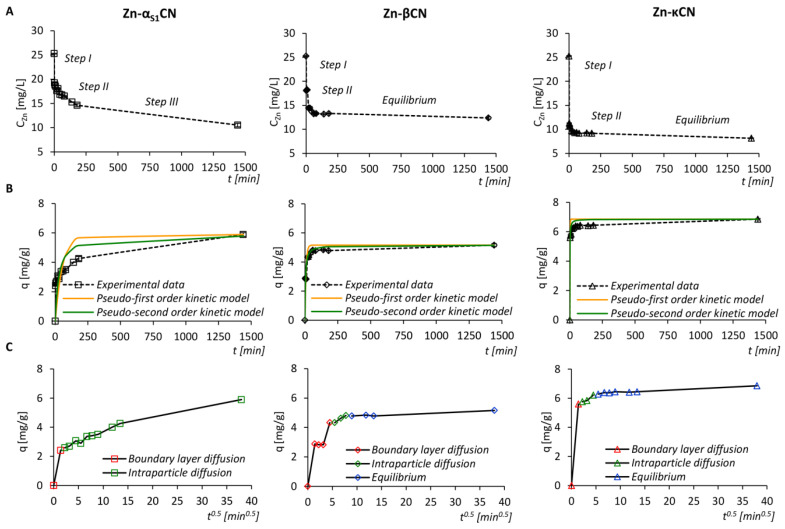
The kinetic steps of the Zn^2+^ sorption onto α_S1_CN, βCN and κCN using the zero-order kinetic model (**A**), experimental data and fitted pseudo-first- and pseudo-second-order kinetics models of the Zn^2+^ sorption by isoforms of casein (**B**) and the Weber–Morris intraparticle diffusion model (**C**).

Figure 2A shows the kinetics of the binding process of zinc ions to casein fractions in the form of a plot of concentration of zinc ions in solution per unit of time. For α_S1_CN, three steps were identified, while for βCN and κCN, two steps were identified. Step I in α_S1_CN, βCN and κCN is associated with rapid initial sorption. Meanwhile, Step II for βCN and κCN is related to slower sorption and gradual achievement of the sorption equilibrium. A different situation is observed in the case of α_S1_CN. Namely, Step II is also associated with gradual sorption, after which Step III appears with an even slower sorption compared to Step II without achieving a state of equilibrium. However, Step II in the case of βCN and κCN is significantly faster compared to α_S1_CN. The obtained results indicate that the process of zinc ion sorption for all three proteins: α_S1_CN, βCN and κCN, is not linear and several separate steps can be identified. 

**Figure 3:**
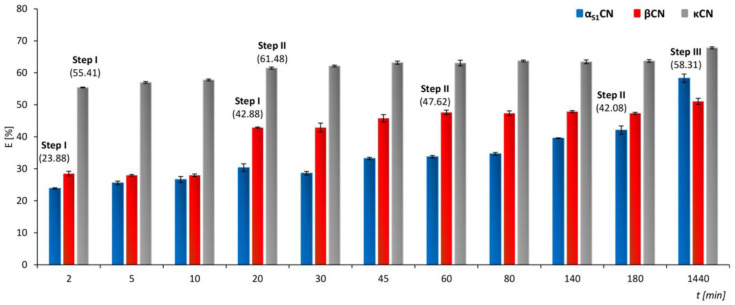
Sorption effectiveness of Zn^2+^ by α_S1_CN, βCN and κCN.

In order to calculate the rate constants of the sorption kinetics of zinc ions for linear segments of the obtained steps, a zero-order kinetics model was used, which describes in detail the successive steps of sorption. The values of the rate constants for Step I in the case of α_S1_CN, βCN and κCN were noticed to be equal to 3.02, 0.54 and 7.0 (mg/L)/min, whereas they were 0.026, 0.030 and 0.085 (mg/L)/min for Step II, respectively. For Step III in the case of α_S1_CN, the rate constant was found to be 0.0033 (mg/L)/min. The results are summarized in Table 1, and in Figure 3, the sorption effectiveness per time unit is shown.

A pseudo-first- and pseudo-second-order kinetic model was applied to the experimental data as well (Figure 2B). The calculated values of the determination coefficient (R^2^) and standard deviation (S) indicated a more accurate description of the kinetics of the sorption of zinc ions to casein proteins by means of a pseudo-second-order kinetics model. The obtained values of the determination coefficient for α_S1_CN and βCN, in comparison to κCN, in the case of both pseudo-second-order kinetics models, indicate low values of the determination coefficient to the obtained experimental results. The not accurate fitting of α_S1_CN and βCN to the classical kinetics model determines the contradictory nature of metal ions sorption in comparison to the glycosylated form of κCN. The calculated kinetic constants are summarized in Table 1. The fast sorption step (Step I) for the two proteins: α_S1_CN and κCN, occurs during the first 2 min of the process, in which 23.88 ± 0.21% of zinc ions were bound to α_S1_CN and the sorption capacity of the protein was 2.41 ± 0.02 mg/g, while for κCN, these values increased slightly and amounted to 55.41 ± 0.13% and 5.60 ± 0.00 mg/g, respectively. For Step I, in the case of βCN, the effectiveness of binding to protein was 42.88 ± 0.20% and the amount of zinc ions absorbed on the protein was 4.33 ± 0.02 mg/g. The sorption process in Step II in the case of α_S1_CN, βCN and κCN ends after 180, 60 and 20 minutes, respectively. The sorption effectiveness of zinc ions by casein isoforms for this step was 42.08 ± 1.31%, 47.62 ± 0.72% and 61.48 ± 0.32%; the sorption capacity was found to be 4.25 ± 0.14, 4.81 ± 0.08 and 6.21 ± 0.05 mg/g. In the last step, Step III, the sorption process for αCN is completed after an incubation period of 1440 min, with a sorption effectiveness of 58.31 ± 1.31% and a sorption capacity of 5.89 ± 0.12 mg/g. The maximum sorption effectiveness and sorption capacity of α_S1_CN, βCN and κCN were 58.31 ± 1.31% and 5.89 ± 0.12 mg/g (α_S1_CN), 51.05 ± 0.97% and 5.16 ± 0.11 mg/g (βCN) and 67.81 ± 0.30% and 6.85 ± 0.05 mg/g (κCN).

In order to determine the mechanism involved in the process of binding zinc ions to casein isoforms, experimental data were also subjected to the Weber–Morris intraparticle diffusion model (Figure 2C). The Weber–Morris model revealed two steps of sorption. Step I was attributed to adsorption on the external surface of proteins or diffusion of zinc ions through the boundary layer as well as a sharp drop in zinc ion concentration. In turn, Step II corresponds to intraparticle diffusion that limits the speed of the process, thus indicating the absorption of zinc ions into the protein structure. In the final step, a sorption equilibrium was noticed. The Weber–Morris model revealed that zinc ions are mainly adsorbed on the external surface of casein isoforms. Then, gradual sorption of zinc ions causes their further diffusion inside the proteins structure.

In addition, the values of the change of Gibbs’ free energy (ΔG^0^) and the distribution coefficient (K_d_) of sorption of zinc ions to α_S1_CN, βCN and κCN were calculated, which were found to be −15.52 kJ/mol and 559.49, −14.80 kJ/mol and 417.25 and −16.52 kJ/mol and 842.62, respectively. Negative values of Gibbs’ free energy for α_S1_CN, βCN and κCN confirm that the process of zinc ion binding to these proteins is spontaneous. The obtained values are presented in Table 1.

The obtained results indicate that at the initial stage of rapid sorption, 1 mole of α_S1_CN, βCN and κCN sorbed 0.86 (α_S1_CN), 1.59 (βCN) and 1.63 (κCN) mole of zinc ions. It turns out that according to the Weber–Morris model, most zinc ions are surface-bound to κCN and the least to α_S1_CN. The situation changes rapidly in a second, slower stage, namely, per one mole of α_S1_CN, βCN and κCN, 2.12, 1.76 and 1.81 mole of zinc ions is sorbed, respectively. This shows that zinc ions are further diffused and absorbed to the greatest extent into the internal structure of α_S1_CN. These results are consistent with the assumptions of the models characterizing casein micelles, where κCN constitutes the outer shell of the micelles, and α_S1_CN is localized in the interior of the casein micelle [14]. Assuming that the synthesis of the binding of zinc ions with individual casein fractions is completed with a state of equilibrium (except for α_S1_CN) after external adsorption steps and penetration of zinc ions into the internal structure of proteins, κCN binding zinc ions on the external surface is the most spontaneous reaction (ΔG^0^ = −16.52 kJ/mol).

### 2.3. Spectroscopic Analysis

**Figure 4:**
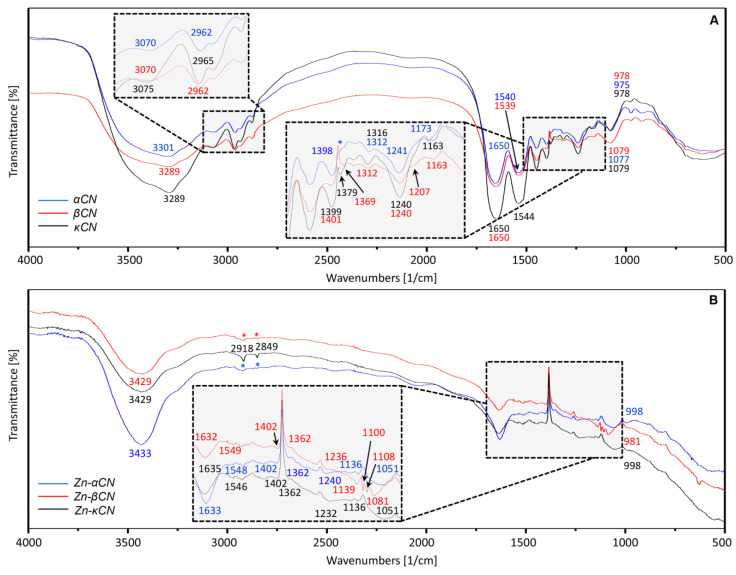
Infrared spectra of the α_S1_CN, βCN and κCN casein isoforms control (**A**) and casein isoforms after binding of zinc ions (**B**). Asterisks (*) indicate signal disappearance.

Spectral characteristics of unmodified (control) α_S1_CN, βCN and κCN as well as the zinc ion-modified ones were determined to establish active chemical groups involved in the zinc ion binding process. The resulting FT-IR spectrum is shown in Figure 4. The obtained FT-IR spectra prove that zinc binding influences the spectrum of individual caseins in infrared. It was observed that after binding zinc to casein, the intensity of selected bands was significantly reduced and the appearance of the spectra changed.

As a result of NH stretching vibrations, the amide band A was observed in the range of 3429–3100 cm^−1^. Its frequency depends on the strength of the hydrogen bond. The amide A band is usually part of Fermi’s resonance double [33]. For unmodified proteins, a shift appeared from 3301 (α_S1_CN) to 3289 cm^−1^ (βCN and κCN). On the other hand, comparing the unmodified proteins (Figure 4A) with Zn-protein complexes (modified, Figure 4B), the shift occurred from the bands of about 3289 to 3429 cm^−1^, respectively.

The spectral range between 3000 and 2840 cm^−1^ is dominated by the absorption of hydrophobic hydrocarbons residue [34]. After binding proteins with zinc ions, bands in the respective region are visible only for the Zn-κCN complex.

The FT-IR spectrum is dominated by the protein amide I (~1650 cm^−1^) and amide II (~1550 cm^−1^) bands, mainly due to the C=O stretching and the NH bending of the peptide bond. The amide I band is extremely sensitive to secondary protein structures (α-helix, β-sheets, random coils) and to the presence of intermolecular β-sheets in protein aggregates [34]. According to Herskovits and co-authors’ [35] studies, the percentage of α-helix in α_S1_CN is estimated at 5–15%, and comparable values of α-helix occur in βCN—7–25%, and κCN—10–20% [4,35]. The percentage of β-sheet in α_S1_CN is between 18 and 20% [36] and 34 and 46% for β-sheet-like [37]. Both α_S1_CN and βCN have around 20–30% turns. The turns are clearly distinguishable from what are commonly called undefined, random or structureless [36]. For βCN, the presence of 15–33% β-sheet has been reported and in the case of κCN, 20–30% of β-structure has been reported [36]. Comparison of the unmodified (control) proteins and zinc-protein complexes for amide I resulted in a shift from the bands of 1650 (control, Figure 4A) to 1633, 1632 and 1635 cm^−1^ (α_S1_CN, βCN and κCN, respectively, Figure 4B). In addition, amide I vibrations, absorbing nearly 1650 cm^−1^, also originate from the stretching vibrations of C=O with a small proportion of CN out-of-phase stretching vibrations, CN deformation and bending in the NH plane. Amide I vibrations are slightly dependent on the nature of the side chain [33]. In amide II, there is an out-of-phase NH in the bend flat, CN stretching vibrations and CN stretching vibrations with less CO in the plane bend and CC and NC stretching vibrations [34]. For amides II, shifts between control proteins and Zn-protein complexes were observed from 1540 (α_S1_CN,), 1539 (βCN) and 1544 (κCN) (Figure 4A) to 1548 (Zn-α_S1_CN), 1549 (Zn-βCN) and 1546 (Zn-κCN) cm^−1^ (Figure 4B). Signals at ~1400 cm^−1^ from amino acid side chains in peptides and proteins are related to CH_3_ asymmetrical and symmetrical bending vibrations [38].The band in this range corresponds to proline (Pro), the amino acid most commonly found both in βCN and κCN, representing 16.7% and 11.8%, respectively, which can make a significant contribution to the binding with zinc ions [33]. However, the bands at ~1540 and ~1400 cm^−1^ may also be assigned to the deprotonated carboxylic groups COO^−^ asparagine (Asp) and glutamine (Glu) residues [39]. The most Glu is contributed by α_S1_CN representing 12.6% of total amino acid residues, being the most common amino acid, followed by βCN at 9.1%, and κCN at 7.1%. Therefore, the binding of zinc ions to α_S1_CN, βCN and κCN can be carried out by coordinating with the oxygen of the side chain Asp and Glu [40,41].

The spectral area 1500–1200 cm^−1^ also includes the area of amide III bands, resulting from bending NH and stretching CN [34]. For the band at about 1401 cm^−1^ corresponding to unmodified βCN, a slight change was observed as opposed to unmodified α_S1_CN and κCN (1398 and 1399 cm^−1^, respectively). However, the bands 1398, 1399 and 1401 cm^−1^, corresponding to unmodified α_S1_CN, κCN and βCN, respectively, after binding to zinc ions are shifted to 1402 cm^−1^ for all proteins. Moreover, for unmodified βCN and κCN, a new band at 1369 cm^−1^ (not see in α_S1_CN) appeared with a slight shift of κCN to 1379 cm^−1^. After binding of proteins to zinc ions, a shift in the 1369 to 1362 cm^−1^ band for βCN was observed and a new 1362 cm^−1^ band for α_S1_CN and κCN was noticed. The bands occurring at ~1240 and ~1079 cm^−1^ are caused by the asymmetrical and symmetrical stretching of ionized PO^2-^, respectively [38]. The control protein bands 1241 (α_S1_CN) and 1240 cm^−1^ (βCN, κCN) were slightly shifted in complexes to 1240 cm^−1^ for α_S1_CN, 1236 cm^−1^ for βCN and 1232 cm^−1^ for κCN. In turn, the bands 1079 (βCN, κCN) and 1077 cm^−1^ (α_S1_CN) were shifted to 1051 (α_S1_CN, κCN) and 1081 cm^−1^ (βCN) after binding to zinc ions. For βCN (unmodified, Figure 4A), a new 1207 cm^−1^ signal was registered, while for α_S1_CN (unmodified) at 1173 cm^−1^, a shift to 1163 cm^−1^ for βCN and κCN (unmodified) was observed. However, comparing these signals (1173, 1163 cm^−1^) with the signals obtained after binding with zinc ions (1136 cm^−1^ for α_S1_CN and κCN, 1139 cm^−1^ for βCN), a shift occurs. For the Zn-βCN complex (Figure 4B), two new 1108 and 1100 cm^−1^ bands were observed. In the case of control proteins (Figure 4A), the bands 975, 978 and 978 cm^−1^ (α_S1_CN, βCN and κCN, respectively) corresponding to the −PO_3_^2−^ moiety of the serine phosphate residue were observed. For complexes (Figure 4B), there was a shift in the bands to a value of about 998 (Zn-α_S1_CN, Zn-κCN) and 981 cm^−1^ (Zn-βCN) to assign the phosphate ions HPO_4_^2-^. The presence of the 978 cm^−1^ band means that CCP (colloidal calcium phosphate) molecules are released from the phosphate residues, causing an increase in the negative charge of casein particles. The appearance of the first band (~978 cm^−1^) suggests that the CCP particle dissociates into Ca^2+^ and HPO_4_^2−^ when the serine phosphate residue is released [42]. In α_S1_CN, there are two phosphorylation centers containing serine (Ser), which is 8.0% and crucial for stabilizing calcium phosphate nanoclusters in casein micelles [4]. These changes may indicate the binding of zinc ions to casein fractions with phosphate ions.

All shifts and appearances of new signals, especially in the range of 1650–978 cm^−1^, occurring in the obtained complexes are correlated with metal–protein binding.

**Figure 5:**
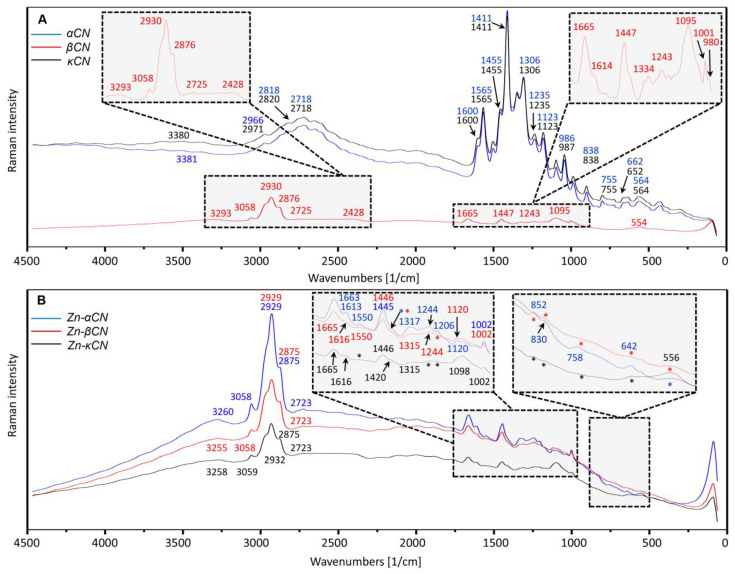
Raman spectra of the α_S1_CN, βCN and κCN casein isoforms control (**A**) and casein isoforms after binding of zinc ions (**B**). Asterisks (*) indicate band disappearance.

Moreover, the use of Raman spectroscopy allowed the observation of vibrational spectra. The Raman spectra provided complementary information to that obtained by IR spectroscopy that showed that apart from carboxylic and phosphate groups, aromatic amino acids also play an important role in metal–protein interaction. The Raman spectra were registered in the 400–4000 cm^−1^ frequency range and are shown in Figure 5.

The Raman protein spectra are dominated by bands associated with the main peptide chain, aromatic side chains and sulphur-containing side chains, and therefore are group vibrations that are either multiple related or electron-rich such as C=O, C=N, C=C, S-S, S-C and S-H. Therefore, changes may result in βCN being similar to complexes rather than to other control proteins [43]. Polar functional groups are characterized by stronger signals in the infrared spectrum, while non-polar functional groups are associated with more intense Raman bands [44].

Figure 5A represents the Raman spectra of unmodified protein fractions. Comparing the spectra for unmodified proteins fractions (α_S1_CN, βCN, κCN), similarities were observed in the case of α_S1_CN and κCN, while βCN indicated large differences in the shape of the registered bands. Raman bands in the range 2428–3381 cm^−1^ were assigned to bond CH (CH_2_, CH_3_), -C-H or =C-H stretch [44]. For the bands 3380 (α_S1_CN) and 3381 cm^−1^ (κCN), a shift was observed for βCN to 3293 cm^−1^ (Figure 5A), while after binding to zinc ions (Figure 5B), a shift was observed to 3260, 3255 and 3258 cm^−1^ for α_S1_CN, βCN and κCN, respectively. For unmodified βCN, the 3058 cm^−1^ band was observed, which is not found in unmodified α_S1_CN and κCN. However, after binding to zinc ions, this band (3058 cm^−1^) is present in all complexes. Shifts were also observed between bands for unmodified proteins—2971 (κCN), 2966 (α_S1_CN) and 2930 cm^−1^ (βCN). After binding of proteins to zinc ions, the absorbance of those bands was similar to unmodified βCN (2929 cm^−1^ for Zn-βCN and Zn-κCN, 2932 cm^−1^ for Zn-α_S1_CN). Similarly, it was observed in the case of bands 2818 and 2718 cm^−1^ (α_S1_CN), 2820 and 2718 cm^−1^ (κCN) and 2876 and 2725 cm^−1^ (βCN) that, after binding, they corresponded to the value of unmodified βCN bands (2875, 2723 cm^−1^ for complexes). 

The spectra registered at amide and amino acids region 500–1655 cm^−1^ illustrate the changes between the modified samples and unmodified. The signals noticed at 1663/1665 cm^−1^ correspond to the C=O stretching mode associated with the CONH protein group (amide I) [45,46], but the bands registered at 1600 and at 1613/1616 cm^−1^ are generated by Tyr-OH [45]. In turn, the 1455 cm^−1^ band is assigned to the NH deformation and CN stretching [46] in amide II. The common bands (1663/1665, 1613/1616, 1550, 1445/1446, 1315/1317, 1244, 1120 and 1002 cm^−1^) have been also noticed for the modified α_S1_CN and βCN protein samples. The bands 1665, 1616, 1446, 1315 and 1002 cm^−1^ were also observed for modified κCN, while bands 1550 and 1244 cm^−1^ for modified κCN disappeared. The 1120 cm^−1^ band corresponding to modified α_S1_CN and βCN for modified κCN has been shifted to 1098 cm^−1^. Disappearance of the band also occurred for unmodified α_S1_CN and κCN at 1665 cm^−1^ and βCN at 1600, 1565 and 1123 cm^−1^. However, the 1411 cm^−1^ band for unmodified α_S1_CN and κCN for unmodified βCN disappeared, and after binding with zinc ions, this band with a shift to 1420 cm^−1^ occurred only for κCN. The mentioned bands, 1002 cm^−1^ for Zn-α_S1_CN, Zn-βCN and Zn-κCN for unmodified proteins, are shifted to 986 (α_S1_CN), 987 (κCN) and 1001 cm^−1^(βCN). The 1002 cm^−1^ band (Figure 5B) corresponds to phenylalanine (Phe) [47]. This amino acid constitutes about 4.0% of the total of residues in α_S1_CN, 4.3% in βCN and 2.4% in κCN and may be responsible for protein binding with zinc ions. The new signals were observed at 1206 and 852 cm^−1^ only for the modified α_S1_CN.

The Raman’s spectra illustrate the amide region III (1200–1340 cm^−1^), including C-N tension and N-H bending [48]. The signal observed at 1306/1315 cm^−1^ is assigned to the alanine (Ala) bands. The band registered at 1235/1244 cm^−1^ corresponds to the CH_2_ carbohydrate twisting mode. The most important current vibration modes have been assigned to CO stretching and deformation of CC and COH (1120–1064 cm^−1^), as well as deformation of COC (950–870 cm^−1^) [46]. What is more, the bands at 852, 830 and 642 cm^−1^ can correspond to the tyrosine (Tyr) vibration [45] and the bands 758 and 556 cm^−1^ to the tryptophan (Trp) [45]. The regions 630–670 cm^−1^ and 700–745 cm^−1^ found in α_S1_CN and κCN control and Zn-α_S1_CN complex originate from C-S stretch cysteine and methionine [44]. The occurrence of residues of Cys11 and Cys88 results in the formation of a complex disulphide bond pattern among κ-CN molecules, with all possible combinations being observed (Cys11-Cys11, Cys11-Cys88 and Cys88-Cys88). There is also a certain amount of monomeric κCN associated with an intramolecular disulphide bond, but it is no more than 10% κCN [49]. In turn, the 564 and 755 cm^−1^ bands are assigned to the tryptophan (Trp) residues [45], which occupy about 1% [4]. Signal shifts in complexes may indicate their participation in the bond with zinc ions.

Similarly, kinetic and spectroscopic studies on the binding of zinc ions to casein carried out by Pomastowski et al. indicate the dominant presence of carboxyl groups Asp and Glu and phosphate groups involved in the binding [9]. In turn, studies on the binding of zinc ions to αCN carried out by Srinivas and Prakash [10] indicate the initial binding to serine phosphates. The binding process is fast, while the affinity of the binding is weak and does not cause any structural changes. However, further reaction with zinc ions leads to binding with aromatic amino acids, including Trp [10]. Zinc binding causes the rearrangement of the secondary protein structure and increases orderliness. It was also observed in the quenching of intrinsic αCN fluorescence by zinc interacting with histidine (His), glutamic acid (Glu), aspartic acid (Asp) and cysteine (Cys) [10]. The presence of Asp and Glu carboxylic groups has also been found in the case of silver to lactoferrin (LTF) binding [23]. The potential binding sites were determined by means of molecular dynamics simulations, which were consistent with and complemented the instrumental studies carried out. For caseins, which are non-crystalline proteins, their total primary and partially secondary structures are known, but homologous proteins with a known crystallographic structure are unavailable. In Kumosiński et al., 1991 [50], an attempt was made to construct a three-dimensional κCN structure using molecular modeling, and the structures obtained were preliminary models. Additionally, despite the fact that the computational approach allows for studying the processes and properties of proteins, there are limitations to the possibility of obtaining accurate parameters that would allow for studying posttranslational modifications (PTMs), which are crucial in caseins’ structure [51]. The proposed casein models require additional validation using other approaches and deserve further investigation, which will be our goal in future studies.

### 2.4. Spectrometric Analysis

**Figure 6:**
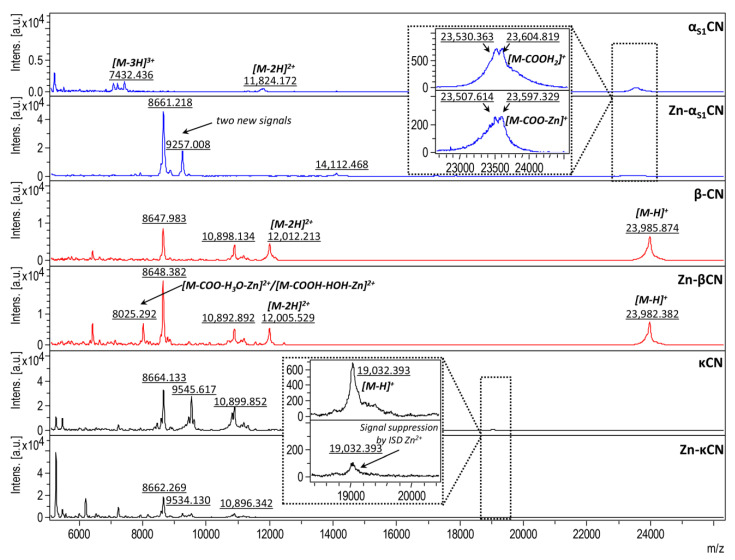
Mass spectra of intact standard solutions of α_S1_CN, βCN, κCN and their complexes with zinc ions (Zn-α_S1_CN, Zn-βCN, Zn-κCN).

Studies on control proteins (unmodified) before binding with zinc ions were performed for three casein fractions obtained during chromatographic separation in our previous studies [52]. To determine the masses of casein isoforms both before and after binding of zinc ions, the analysis of intact proteins was carried out using MALDI-TOF MS (Figure 6).

The masses of intact casein isoforms were found to be 23,985.874 ± 0.326 and 19,032.393 ± 0.326 m/z for βCN and κCN, respectively. In the case of α_S1_CN, two overlapping signals were observed. These signals correspond to values 23,530.363 ± 0.326 and 23,604.819 ± 0.326 m/z. According to the literature data, the registered signals are coming from different genetic forms of α_S1_CN [53,54].

The respective signals appeared in the sample after zinc ions binding (Zn-α_S1_CN complex) at 23,507.614 and 23,597.329 m/z. This difference is connected to the degradation of some parts of the protein structure during the zinc ions binding process. Compared to the unmodified protein (control), in the case of the Zn-α_S1_CN complex, a new signal has been observed (8661.218 and 9257.008 m/z). This observation is the result of protein decay to the more hydrophobic fragments, e.g., 8661.218 and 9257.008 m/z (Figure 6). A different situation was observed in the case of the βCN protein. The characteristic signal registered at 23,985.874 ± 0.326/23 and 982.382 ± 0.326 m/z was noticed in both the unmodified and modified βCN protein. Moreover, in the case of the Zn-βCN complex, a new signal with the m/z ratio of 8025.292 m/z has been noticed. This signal indicates that hydrophilic zinc ions bind to the hydrophobic protein through indirect interaction with oxygen from water. βCN is more hydrophobic than other casein isoforms. This is due to a negative net charge in the N-terminal with five phosphoserylic and hydrophobic C-terminal residues [55]. In the case of the κCN protein, which is the smallest of the caseins, the signal is suppressed (in comparison with the control signal 19,032.393 m/z) after binding to the zinc ions. Perhaps it is an effect of glocosylation occurring only in κCN. The κCN is a glycoprotein sensitive to the proteinase (chymosin) site, which causes cleavage of the glycoprotein into two parts: the N-terminal para- κ-casein domain and the C-terminal domain of κ-casein with macroglyceride, which is highly heterogeneous in terms of oligosaccharide content [56]. 

## 3. Materials and Methods

### 3.1. Characteristics of α_S1_CN, βCN and κCN

#### 3.1.1. Isolation of α_S1_CN, βCN and κCN, Chromatographic Separation and Matrix-Assisted Laser Desorption Ionization−Time of Flight Mass Spectrometry (MALDI-TOF/TOF-MS) Analysis

The casein fractions (α_S1_CN, βCN, κCN) used in present study have been previously separated and identified by our research group according to Pomastowski et al. [52] using high-performance liquid chromatography (HPLC) and MALDI-TOF MS techniques, respectively. In the current research, the isolated fractions were used to continue the study by the investigation of the mechanism of binding zinc ions with the obtained casein fractions. 

#### 3.1.2. Isoelectric Point Determination

The isoelectric point of casein isolated from milk was determined by the diffraction light scattering technique (Zetasizer, Malvern Instruments, Malvern, UK). The protein was suspended in 0.09% sodium chloride solution (Sigma-Aldrich, Warszawa, Poland) in the range of pH 2–11, sonicated for 10 s and analyzed using the DTS1070 cuvette (Malvern Panalitycal). All the measurements were performed in three repetitions.

### 3.2. Kinetic Study of Zinc Ions Binding to α_S1_CN, βCN and κCN

For the kinetic studies, the samples were prepared by mixing α_S1_CN, βCN, κCN and zinc nitrate (V) (Sigma-Aldrich, Warszawa, Poland) solutions at a ration of 1:1 (*v*/*v*) with the final concentration of 5000 and 25 mg/L. The protein samples were suspended in 0.09% sodium chloride at pH = pI. Then, the samples were incubated at 4 °C and analyzed after a certain period of time: 2, 5, 10, 20, 30, 45, 60, 80, 140, 180 and 1440 min, and centrifuged (12,000 rpm, 10 min). Part of the supernatant was mineralized in aqua regia and diluted to 1% nitric acid (V) (Sigma Aldrich, Poland). The concentration of zinc ions was determined by inductively coupled plasma-mass spectrometry ICP-MS (7900 ICP-MS, Agilent Technologies).

To explain the mechanism of zinc ion sorption by casein isoforms, zero kinetics, pseudo-first- and pseudo-second-order kinetics models, and intraparticle diffusion models were used to analyze the results.

The kinetic models were expressed with the following formulas:

- The zero-order kinetics model:

(1) $$C=\;C_{0}-k_{0}t$$

where: *C*—the concentration of zinc ions in aqueous solution for a certain period of time [mg/L], *C*_0_—the initial concentration of zinc ions in aqueous solution [mg/L], *k*_0_—the adsorption rate constant [(mg/L)/min], and *t*—the adsorption duration [min].

- The pseudo-first-order kinetics model:

(2) $$q_{t}=q_{e}\left(1-e^{-k_{1}t}\right)$$

where: *q_t_*—the amount of zinc ions sorbed for a certain period of time [mg/g], *q_e_*—the amount of zinc sorbed at equilibrium [mg/g], and *k*_1_—the rate constant of the pseudo-first-order sorption kinetics [1/min].

- The pseudo-second-order kinetics model:

(3) $$q_{t}=\frac{q_{e}^{2}k_{2}t}{1+q_{e}k_{2}t}$$

where: *k*_2_—the rate constant of the pseudo-second-order sorption kinetics [(g/mg)/min].

- The Weber–Morris intraparticle diffusion model:

(4) $$q_{t}=A+K_{ip}t^{0.5}$$

where: *A*—a constant indicating the thickness of the boundary layer diffusion or external surface adsorption [mg/g], and *K_ip_*—the intraparticle diffusion rate constant [(mg/g)/min^0.5^] 

The amount of zinc sorption by casein isoforms from an aqueous solution (for experimental data) was determined as follows:

(5) $$q_{t}=\left(C_{0}-C\right)\frac{V}{m}$$

where: *V*—the volume of solution from which sorption occurs [L], and *m*—the sorbent mass [g].

Additionally, the distribution coefficient (*K_d_*) of zinc ions sorption by α_S1_CN, βCN and κCN and Gibbs’ free energy for zinc adsorption were calculated.

The following equations were used:

(6) $$K_{d}=\frac{q_{e}}{C_{e}}$$

where: *q_e_*—the amount of zinc sorbed by casein isoforms at equilibrium time [mg/g], and *C_e_*—the equilibrium concentrations of zinc in solution [mg/L].

(7) $$∆G^{0}=-RTlnK_{d}$$

where: ∆*G*^0^—the energy of adsorption [kJ/mol], *R*—the gas constant (8.314 J/mol·K), *T*—the adsorption absolute temperature (295 K), and *K_d_*—the distribution coefficient.

### 3.3. Spectroscopic and Spectrometric Analysis

For the FT-IR, Raman and MALDI-TOF MS studies, the complexes were prepared by mixing α_S1_CN, βCN, κCN and zinc nitrate (V) (Sigma-Aldrich, Warszawa, Poland) solutions at a ration of 1:1 (*v*/*v*) with the final concentrations of 5000 and 25 mg/L. The protein samples were suspended in 0.09% sodium chloride at pH = pI. Then, the samples were incubated at 4 °C for 24 h (because, after this time, we are sure that the metal–protein binding will take place). After the incubation time, the samples were centrifuged (12,000 rpm, 10 min). The supernatant was removed and the resulting sediment was lyophilized and submitted to further spectroscopic and spectrometric analyses. In turn, isolated CN (control) was prepared by dissolving in distilled water.

#### 3.3.1. Fourier Transform Infrared Spectroscopic (FT-IR) and Raman Spectroscopy (Raman) Analysis of α_S1_CN, βCN and κCN

Spectroscopic methods (FT-IR, Raman) have been used as techniques for analyzing the changes of the active functional group and the secondary structure of the protein system. 

Spectra in FT-IR analysis were recorded by using a Spectrum 2000 from Perkin-Elmer, Waltham, MA, and were recorded in the MIR range from 400 to 4000 cm^−1^, and 15 scans were averaged at a resolution of 4. The samples were prepared in three repetitions by grinding them with potassium bromide powder (KBr) and pressed into a disc.

The Raman signals were recorded with the Raman Spectrometer (Senterra, Bruker Optik) in a spectral range of 400–4000 cm^−1^ with an integration time of 3 × 30 s using a 532 nm laser excitation, at 20 × 5 mW power, in combination with 10 fM accumulation. The samples were prepared in three repetitions by direct application of the sample on a microscope slide.

All spectra were processed using ORIGIN software.

#### 3.3.2. Matrix-Assisted Laser Desorption Ionization−Time of Flight Mass Spectrometry (MALDI-TOF/TOF-MS) Analysis before and after Zing Binding to Isoforms of Casein

MALDI mass spectra were acquired using a Bruker UltrafleXtreme II mass spectrometer provided with 2 kHz speed in TOF mode and 1 kHz speed in TOF/TOF mode equipment with a modified Nd:YAG laser operating at the wavelength of 355 nm. For MALDI-TOF MS analysis of the intact proteins before and after binding of zinc to isoforms of casein, samples were prepared according to the dried droplet method using sinapinic acid (SA) as matrix, and next, the samples were applied to ground steel. In turn, Protein Calibration Standards II was selected for calibration. Spectra were obtained in linear positive ion mode over an *m/z* range of 5000–100,000.

## 4. Conclusions

This study describes for the first time the mechanism of binding zinc ions to individual casein fractions, causing the formation of complexes, and thus indicating the precise contribution of individual fractions to the binding. The performed kinetic studies of the binding of zinc ions with α_S1_CN, βCN and κCN indicate a heterogeneous kinetic process carried out in three steps for Zn-α_S1_CN, Zn-βCN and Zn-κCN. The initial stage is associated with rapid initial sorption, the second stage with moderate sorption and the third stage with gradual achievement of the sorption equilibrium. Experimental data subjected to the Weber–Morris model indicated two stages of sorption ending in a sorption equilibrium step. The first step was related to adsorption occurring on the external surface of proteins, while the second step was related to intraparticle diffusion of zinc ions. 

Spectroscopic studies (FT-IR) have proven that the main role in the binding of zinc ions to α_S1_CN, βCN and κCN complexes is played phosphate groups and carboxylic groups of Glu and Asp. Raman’s spectroscopy supplemented the information from the gap in the information obtained from FT-IR analysis and indicates that the presence of aromatic amino acids such as Tyr, Trp and Phe was involved in the binding with zinc ions.

The use of mass spectrometry allows the accurate determination of masses and thus the identification of α_S1_CN, βCN and κCN. The mass spectra obtained after binding the protein with zinc ions indicate that in the case of α_S1_CN, the carboxylic groups Asp and Glu play a key role. βCN binds to zinc ions through indirect interactions with oxygen ions, whereas in κCN, the binding of zinc ions takes place probably through weak electrostatic interactions with deprotonated functional groups. However, the proposed binding mechanism requires additional validation using complementary approaches, especially molecular modeling methods.

## Figures and Tables

**Table 1:** Kinetic model parameters for the zinc ions sorption by α_S1_CN, βCN and κCN and values of the distribution coefficient and the Gibbs’ free energy change of the metal ions sorption.

		Zn-α_S1_CN	Zn-βCN	Zn-κCN
**Zero-order kinetic model**	k_0_ [(mg/L)/min]	3.02	0.54	7.00
		0.026	0.030	0.085
		0.0033		
**Pseudo-first-order kinetic model**	k_1_ [1/min]	0.018	0.13	0.76
	S	1.32	0.70	0.58
	R^2^	0.075	0.78	0.90
**Pseudo-second-order kinetic model**	k_2_ [(g/mg)/min]	0.0065	0.050	0.21
	S	1.02	0.80	0.40
	R^2^	0.45	0.71	0.96
**Intraparticle diffusion model**	A [mg/g]	2.58	3.56	5.30
	K_ip_ [(mg/g)/min^−0.5^]	0.094	0.16	0.19
	S	0.25	0.083	0.066
	R^2^	0.94	0.92	0.96
**Distribution coefficient, the Gibbs’ free energy change of the metal ions sorption**	q_e_ [mg/g]	5.89	5.16	6.85
	C_e_ [mg/L]	10.53	12.37	8.13
	K_d_	559.49	417.25	842.62
	T [K]	295	295	295
	ΔG^0^ [kJ/mol]	−15.52	−14.80	−16.52

K_ip_—the intraparticle diffusion rate constant; q_e_—the amount of zinc sorbed by casein isoforms at equilibrium time; C_e_—the equilibrium concentrations of zinc in solution; K_d_—the distribution coefficient of zinc ions sorption by α_S1_CN, βCN and κCN.

## Abbreviations

α_S1_CN	α_S1_-casein
βCN	β-casein
κCN	κ-casein
Ala	Alanine
Asp	Aspartic acid
Glu	Glutamic acid
Phe	Phenylalanine
Pro	Proline
Thr	Threonine
Trp	Tryptophan
Tyr	Tyrosine
Ser	Serine
CCP	Colloidal calcium phosphate
FT-IR	Fourier transform infrared spectroscopy
HPLC	High-performance liquid chromatography
MALDI-TOF MS	Matrix-assisted laser desorption/ionization time-of-flight mass spectrometry

## References

[1] Bhat M.Y., Dar T.A., Singh L.R. (2016). Casein Proteins: Structural and Functional Aspects. Milk Proteins—From Structure to Biological Properties and Health Aspects.

[2] Imafidon G.I., Farkye N.Y., Spanier A.M. (1997). Isolation, purification, and alteration of some functional groups of major milk proteins: A review. Crit. Rev. Food Sci. Nutr..

[3] Müller-Buschbaum P., Gebhardt R., Roth S.V., Metwalli Z.E., Doster W. (2007). Effect of calcium concentration on the structure of casein micelles in thin films. Biophys. J..

[4] Huppertz T., Fox P.F., Kelly A.L. (2018). The Caseins: Structure, Stability, and Functionality.

[5] Glantz M., Devold T.G., Vegarud G.E., Lindmark Månsson H., Stålhammar H., Paulsson M. (2010). Importance of casein micelle size and milk composition for milk gelation. J. Dairy Sci..

[6] Pérès J.M., Bouhallab S., Petit C., Bureau F., Maubois J.L., Arhan P., Bouglé D. (1998). Improvement of zinc intestinal absorption and reduction of zinc/iron interaction using metal bound to the caseinophosphopeptide 1-25 of β-casein. Reprod. Nutr. Dev..

[7] Pryshchepa O., Sagandykova G.N., Pomastowski P., Railean-Plugaru V., Król A., Rogowska A., Rodzik A., Sprynskyy M., Buszewski B. (2019). A New Approach for Spontaneous Silver Ions Immobilization onto Casein. Int. J. Mol. Sci..

[8] Demott B.J., Dincer B. (1976). Binding Added Iron to Various Milk Proteins. J. Dairy Sci..

[9] Pomastowski P., Sprynskyy M., Buszewski B. (2014). The study of zinc ions binding to casein. Colloids Surf. B Biointerfaces.

[10] Srinivas S., Prakash V. (2011). Interaction of Zn (II) with bovine milk α-casein: Structure-function study. J. Food Biochem..

[11] Farrell H.M. (1973). Models for Casein Micelle Formation. J. Dairy Sci..

[12] Broyard C., Gaucheron F. (2015). Modifications of structures and functions of caseins: A scientific and technological challenge. Dairy Sci. Technol..

[13] Głąb T.K., Boratyński J. (2017). Potential of Casein as a Carrier for Biologically Active Agents. Top. Curr. Chem..

[14] Hristov P., Mitkov I., Sirakova D., Mehandgiiski I., Radoslavov G. (2016). Measurement of Casein Micelle Size in Raw Dairy Cattle Milk by Dynamic Light Scattering. Milk Proteins—From Structure to Biological Properties and Health Aspects.

[15] Morr C.V. (1967). Effect of Oxalate and Urea upon Ultracentrifugation Properties of Raw and Heated Skimmilk Casein Micelles. J. Dairy Sci..

[16] Waugh D.F., Noble R.W. (1965). Casein Micelles. Formation and Structure. J. Am. Chem. Soc..

[17] Rose D. (1968). Relation Between Micellar and Serum Casein in Bovine Milk. J. Dairy Sci..

[18] Wilkins T.D., Velander W. (1992). Isolation of recombinant proteins from milk. J. Cell. Biochem..

[19] Donnelly W.J. (1977). Chromatography of milk proteins on hydroxyapatite. J. Dairy Res..

[20] Yang X., Jiang L., Jia Y., Hu Y., Xu Q., Xu X., Huang H. (2016). Counteraction of trehalose on N, N-dimethylformamide-induced Candida rugosa lipase denaturation: Spectroscopic insight and molecular dynamic simulation. PLoS ONE.

[21] Rouhier N., Gelhaye E., Jacquot J.P. (2002). Glutaredoxin-dependent peroxiredoxin from poplar. Protein-protein interaction and catalytic mechanism. J. Biol. Chem..

[22] Zänker H., Heine K., Weiss S., Brendler V., Husar R., Bernhard G., Gloe K., Henle T., Barkleit A. (2019). Strong Uranium(VI) Binding onto Bovine Milk Proteins, Selected Proteins Sequences, and Model Peptides. Inorg. Chem..

[23] Pomastowski P., Sprynskyy M., Žuvela P., Rafińska K., Milanowski M., Liu J.J., Yi M., Buszewski B. (2016). Silver-Lactoferrin Nanocomplexes as a Potent Antimicrobial Agent. J. Am. Chem. Soc..

[24] Dokmanić I., Šikić M., Tomić S. (2008). Metals in proteins: Correlation between the metal-ion type, coordination number and the amino-acid residues involved in the coordination. Acta Crystallogr. Sect. D Biol. Crystallogr..

[25] Miki T., Awa M., Nishikawa Y., Kiyonaka S., Wakabayashi M., Ishihama Y., Hamachi I. (2016). A conditional proteomics approach to identify proteins involved in zinc homeostasis. Nat. Methods.

[26] Watt N.T., Taylor D.R., Kerrigan T.L., Griffiths H.H., Rushworth J.V., Whitehouse I.J., Hooper N.M. (2012). Prion protein facilitates uptake of zinc into neuronal cells. Nat. Commun..

[27] Kitamura H., Morikawa H., Kamon H., Iguchi M., Hojyo S., Fukada T., Yamashita S., Kaisho T., Akira S., Murakami M. (2006). Toll-like receptor-mediated regulation of zinc homeostasis influences dendritic cell function. Nat. Immunol..

[28] Harzer G., Kauer H. (1982). Binding of zinc to casein. Am. J. Clin. Nutr..

[29] Pomastowski P.P., Dziubakiewicz E., Buszewski B. (2012). Potencjał zeta—jego rola i znaczenie. Analityka.

[30] Roosen-Runge F., Heck B.S., Zhang F., Kohlbacher O., Schreiber F. (2013). Interplay of pH and binding of multivalent metal ions: Charge inversion and reentrant condensation in protein solutions. J. Phys. Chem. B.

[31] Farrell H.M., Jimenez-Flores R., Bleck G.T., Brown E.M., Butler J.E., Creamer L.K., Hicks C.L., Hollar C.M., Ng-Kwai-Hang K.F., Swaisgood H.E. (2004). Nomenclature of the proteins of cows’ milk—Sixth revision. J. Dairy Sci..

[32] Egito A.S., Miclo L., López C., Adam A., Girardet J.M., Gaillard J.L. (2002). Separation and characterization of mares’ milk αs1-, β-, κ-caseins, γ-casein-like, and proteose peptone component 5-like peptides. J. Dairy Sci..

[33] Barth A. (2007). Infrared spectroscopy of proteins. Biochim. Biophys. Acta Bioenerg..

[34] Ami D., Lavatelli F., Rognoni P., Palladini G., Raimondi S., Giorgetti S., Monti L., Doglia S.M., Natalello A., Merlini G. (2016). In situ characterization of protein aggregates in human tissues affected by light chain amyloidosis: A FTIR microspectroscopy study. Sci. Rep..

[35] Herskovits T.T. (1966). On the Conformation of Caseins. Optical Rotatory Properties. Biochemistry.

[36] Michael Byler D., Farrell H.M., Susi H. (1988). Raman Spectroscopic Study of Casein Structure. J. Dairy Sci..

[37] Malin E.L., Brown E.M., Wickham E.D., Farrell H.M. (2005). Contributions of terminal peptides to the associative behavior of αs1-casein. J. Dairy Sci..

[38] Cestelli Guidi M., Mirri C., Fratini E., Licursi V., Negri R., Marcelli A., Amendola R. (2012). In vivo skin leptin modulation after 14 MeV neutron irradiation: A molecular and FT-IR spectroscopic study. Anal. Bioanal. Chem..

[39] Parikh S.J., Kubicki J.D., Jonsson C.M., Jonsson C.L., Hazen R.M., Sverjensky D.A., Sparks D.L. (2011). Evaluating Glutamate and Aspartate Binding Mechanisms to Rutile (α-TiO_2_ ) via ATR-FTIR Spectroscopy and Quantum Chemical Calculations. Langmuir.

[40] Ryde U. (1999). Carboxylate binding modes in zinc proteins: A theoretical study. Biophys. J..

[41] Krężel A., Maret W. (2016). The biological inorganic chemistry of zinc ions. Arch. Biochem. Biophys..

[42] Gebhardt R., Takeda N., Kulozik U., Doster W. (2011). Structure and stabilizing interactions of casein micelles probed by high-pressure light scattering and FTIR. J. Phys. Chem. B.

[43] Thomas G.J. (1999). Raman spectroscopy of protein and nucleic acid assemblies. Annu. Rev. Biophys. Biomol. Struct..

[44] Li-Chan E.C.Y. (2007). Vibrational spectroscopy applied to the study of milk proteins. Dairy Sci. Technol..

[45] Li-Chan E., Nakai S., Hirotsuka M. (1994). Raman spectroscopy as a probe of protein structure in food systems. Protein Structure-Function Relationships in Foods.

[46] Almeida M.R., Oliveira K.D.S., Stephani R., De Oliveira L.F.C. (2011). Fourier-transform Raman analysis of milk powder: A potential method for rapid quality screening. J. Raman Spectrosc..

[47] Jarvis R.M., Blanch E.W., Golovanov A.P., Screen J., Goodacre R. (2007). Quantification of casein phosphorylation with conformational interpretation using Raman spectroscopy. Analyst.

[48] Kurouski D., Van Duyne R.P., Lednev I.K. (2015). Exploring the structure and formation mechanism of amyloid fibrils by Raman spectroscopy: A review. Analyst.

[49] Farrell H.M., Kumosinski T.F., Cooke P.H., King G., Hoagland P.D., Wickham E.D., Dower H.J., Groves M.L. (1996). Particle sizes of purified κ-casein: Metal effect and correspondence with predicted three-dimensional molecular models. J. Protein Chem..

[50] Kumosinski T.F., Brown E.M., Farrell H.M. (1991). Three-Dimensional Molecular Modeling of Bovine Caseins: κ-Casein. J. Dairy Sci..

[51] Audagnotto M., Dal Peraro M. (2017). Protein post-translational modifications: In silico prediction tools and molecular modeling. Comput. Struct. Biotechnol. J..

[52] Pomastowski P., Walczak J., Gawin M., Bocian S., Piekoszewski W., Buszewski B. (2014). HPLC separation of casein components on a diol-bonded silica column with MALDI TOF/TOF MS identification. Anal. Methods.

[53] Vincent D., Elkins A., Condina M.R., Ezernieks V., Rochfort S. (2016). Quantitation and identification of intact major milk proteins for high-throughput LC-ESI-Q-TOF MS analyses. PLoS ONE.

[54] Mamone G., Caira S., Garro G., Mauriello R., Nicolai M.A., Picariello G., Calabrese M.G., Ferranti P., Chianese L., Addeo F. (2013). Challenging the heterogeneity of casein by an IEF/MALDI-TOF “virtual 2D-like” approach. Food Res. Int..

[55] Wong D.W.S., Camirand W.M., Pavlath A.E. (1996). Structures and Functionalities of Milk Proteins.

[56] Pisano A., Packer N.H., Redmond J.W., Williams K.L., Gooley A.A. (1994). Characterization of O-linked glycosylation motifs in the glycopeptide domain of bovine κ-casein. Glycobiology.

